# Association of Childhood Family Connection With Flourishing in Young Adulthood Among Those With Type 1 Diabetes

**DOI:** 10.1001/jamanetworkopen.2020.0427

**Published:** 2020-03-05

**Authors:** Robert C. Whitaker, Tracy Dearth-Wesley, Allison N. Herman, Kathryn E. Nagel, Hannah G. Smith, Henry F. C. Weil

**Affiliations:** 1 Columbia-Bassett Program, Vagelos College of Physicians and Surgeons, Columbia University, New York, New York; 2 Columbia-Bassett Program, Bassett Medical Center, Cooperstown, New York; 3 Bassett Research Institute, Bassett Medical Center, Cooperstown, New York; 4 Vagelos College of Physicians and Surgeons, Department of Pediatrics, Columbia University, New York, New York; 5 College of Public Health, Temple University, Philadelphia, Pennsylvania; 6 now with School of Medicine, Yale University, New Haven, Connecticut; 7 now with College of Medicine, SUNY Upstate Medical University, Syracuse, New York; 8 Vagelos College of Physicians and Surgeons, Department of Medicine, Columbia University, New York, New York

## Abstract

**Importance:**

Higher levels of childhood family connection have been associated with measures of adult flourishing or eudaimonic well-being, such as purpose, self-acceptance, positive relationships, and growth. However, this association has not been examined among those with childhood-onset chronic disease.

**Objectives:**

To investigate whether higher levels of childhood family connection were associated with greater flourishing in young adulthood among those with type 1 diabetes and, secondarily, whether this association was present across levels of adverse childhood experiences and childhood social position.

**Design, Setting, and Participants:**

In 2017, the cross-sectional Type 1 Flourish survey was administered to all 743 young adults, aged 18 to 29 years, with type 1 diabetes who had received outpatient care in 2016 at a diabetes specialty clinic in New York, New York. Eligible participants completed the survey online or during clinic visits. Data analyses were conducted in September and October 2019.

**Exposures:**

The main exposure was childhood family connection (sample-defined tertiles), based on scores from a 7-item scale assessing parental attention, affection, and communication during childhood. Adverse childhood experiences, childhood social position, and other sociodemographic characteristics were also reported. Recent hemoglobin A_1c_ levels were abstracted from medical records.

**Main Outcomes and Measures:**

Flourishing score calculated from the 42-item Psychological Well-being Scale developed by Ryff.

**Results:**

The survey was completed by 423 of 743 patients (56.9%), and the analysis included 415 participants (98.1%) with complete data on family connection and flourishing. The mean (SD) age of the sample was 25.0 (3.2) years, with 246 (59.3%) female respondents and 288 (69.6%) non-Hispanic white respondents. The mean (SD) flourishing score was 221.8 (37.7). After adjusting for age, sex, race/ethnicity, education, income, age at type 1 diabetes diagnosis, and hemoglobin A_1c_ level, mean flourishing scores increased from the lowest (201.0; 95% CI, 195.0-207.0) to medium (225.2; 95% CI, 219.4-231.0) to highest (240.4; 95% CI, 234.4-246.4) tertiles of family connection; compared with those in the lowest tertile of family connection, the flourishing scores were 1.04 (95% CI, 0.81-1.27) SD units higher among those in the highest tertile and 0.64 (95% CI, 0.42-0.86) SD units higher among those in the middle tertile. This association was also present across levels of childhood adversity. In the subgroup of respondents with 2 or more adverse childhood experiences, those in the highest tertile of family connection had adjusted flourishing scores 0.76 (95% CI, 0.14-1.38) SD units higher than those in the lowest tertile. In the subgroup with low childhood social position, those in the highest tertile of family connection had flourishing scores 1.08 (95% CI, 0.63-1.52) SD units higher than those in the lowest tertile.

**Conclusions and Relevance:**

In this cross-sectional study of young adults with type 1 diabetes, higher levels of childhood family connection were associated with greater flourishing in young adulthood across levels of childhood adversity. Beyond disease management, clinician support of family connection may help children with type 1 diabetes flourish in adulthood.

## Key Points

**Question:**

Is family connection during childhood associated with greater levels of flourishing in young adulthood among those with type 1 diabetes?

**Findings:**

This cross-sectional study included 415 young adults with type 1 diabetes. After adjusting for glycemic control, there was a significant graded association of childhood family connection with adult flourishing, and this association was seen across levels of adverse childhood experiences and childhood social position.

**Meaning:**

Across levels of childhood adversity, greater childhood family connection was associated with greater flourishing among young adults with type 1 diabetes.

## Teaser

This cross-sectional study investigates whether levels of childhood family connection are associated with greater flourishing in young adulthood among those with type 1 diabetes and whether this association is present across levels of adverse childhood experiences and childhood social position.

## Introduction

Higher levels of psychological well-being are associated with greater functioning and longevity among adults who have chronic physical or mental health conditions.^1,2,3,4^ Because disease and disability are inevitable across the life course, entering adulthood with high psychological well-being may allow adults to adapt more successfully to aging.^5^ This is especially true among those with childhood-onset chronic health conditions, such as type 1 diabetes.^6^

Adult psychological well-being has many dimensions,^7,8,9^ and its subjective evaluation is based on 2 primary frameworks.^10,11,12^ The hedonic framework considers the experience of emotion, both positive and negative, with a focus on happiness and the evaluation of life satisfaction. In contrast, the eudaimonic framework, which we here call *flourishing*, describes positive mental functioning, with a focus on having a sense of meaning and purpose in life through the awareness and realization of one’s potential and limitations.^12^

Flourishing does not require or exclude the experience of happiness; rather, it allows for the ubiquitous nature of adversity, not all of which is preventable.^13^ Some adversities in childhood, such as abuse, neglect, poverty, and chronic illness, can be traumatic if they result in emotional experiences that have lasting effects on functioning.^14^ Integrating adversity into the concept of flourishing makes it especially relevant for pediatric practice, which addresses childhood-onset chronic disease in the context of other challenges.^15,16,17^

There is emerging evidence that childhood family connection is associated with greater flourishing in adulthood.^18,19,20,21,22,23^ In these studies, family connection was characterized by the type of attention, affection, and communication that creates safe, stable, and nurturing relationships between adult caregivers and children.^24,25^ Many studies have documented that measures of parent-child relationship quality, from parental warmth and responsiveness to secure attachment, are associated with positive adult outcomes. However, these successful outcomes are often characterized as the avoidance of negative events or conditions, particularly following childhood adversity, rather than as the attainment of flourishing.^26,27,28,29^ In a cross-sectional study of US children, family connection was associated with a measure of child flourishing, and this association was found across levels of adversity.^30^ However, we are unaware of any studies that have assessed the association of childhood family connection with adult flourishing among those with childhood-onset chronic illness, nor to our knowledge, have any studies demonstrated that childhood family connection is associated with adult flourishing across levels of childhood adversity.

Using data from a cross-sectional survey of young adults with type 1 diabetes, we investigated whether childhood family connection was associated with greater flourishing in young adulthood. We also examined whether this association was present across levels of adverse childhood experiences (ACEs) and childhood social position.

## Methods

In the spring of 2017, the online survey Type 1 Flourish: Strengths and Challenges for Young Adults With Type 1 Diabetes was administered to young adults with type 1 diabetes receiving care at the Naomi Berrie Diabetes Center (NBDC) at the Columbia University Irving Medical Center in New York, New York. The purpose of this survey was to examine the associations between glycemic control, aspects of positive psychological well-being, and adversities, both past and present. The original and present study were approved by the institutional review boards at Columbia University and Temple University. This report follows the Strengthening the Reporting of Observational Studies in Epidemiology (STROBE) reporting guideline. Data analyses were conducted in September and October 2019.

### Participants and Procedures

In the eAppendix in the Supplement, we provide details about the survey procedures, which are summarized here. Eligible survey participants had a diagnosis of type 1 diabetes, had at least 1 outpatient visit to NBDC between December 1, 2015, and November 30, 2016, and were aged 18 years to younger than 30 years on December 1, 2015. The 746 patients identified through electronic medical records were reviewed by NBDC physicians treating patients with type 1 diabetes. Three patients were excluded because of cognitive impairments that prevented them from comprehending the survey. The 743 eligible patients were recruited via email, posted mail, and through the NBDC website and clinic. After providing authenticating information online, participants provided written informed consent before beginning the survey. Of the 743 eligible patients, 423 patients (56.9%) completed the survey. Participants were compensated $20 for completing the survey.

### Measures

#### Primary Exposure: Childhood Family Connection

Childhood family connection was measured using 7 items from the Midlife in the United States (MIDUS) Study.^31,32^ This set of items was asked separately about each parent, asking the respondent to consider “the mother/father (or the woman/man who raised you) during the years you were growing up.” The first item asked, “How would you rate your relationship with your mother/father?” Five responses were provided, ranging from excellent (1) to poor (5). The remaining items addressed parental attention, affection, and communication. Survey respondents were asked to rate the following 6 characteristics of the parent: (1) “How much did she/he understand your problems and worries?” (2) “How much could you confide in her/him about things that were bothering you?” (3) “How much love and affection did she/he give you?” (4) “How much time and attention did she/he give you when you needed it?” (5) “How much effort did she/he put into watching over you and making sure you had a good upbringing?” and (6) “How much did she/he teach you about life?” For these 6 items, respondents were provided the 4 following rating options: a lot (1), some (2), a little (3), and not at all (4).

Item scores were recoded so that higher scores reflected greater levels of connection. Separate maternal and paternal connection scores were calculated as the mean score of the 7 items, with the first item score (range, 1-5) multiplied by 0.75 to align with the other 6 items (range, 1-4).^22,32^ Consistent with the approach of others,^19,22,23^ a childhood family connection score was determined by averaging the maternal and paternal connection scores. The internal consistency (Cronbach α) of the family connection score and both the maternal and paternal subconstruct scores were at least 0.90 (eTable 1 in the Supplement). To facilitate clinical interpretation of the magnitude and relevance of the association of family connection with flourishing, family connection was analyzed in 3 groups based on sample tertiles, as follows: low, less than 3.13; medium, 3.13 to 3.70; and high, greater than 3.70.

#### Primary Outcome: Adult Flourishing

Flourishing was measured using the Psychological Well-being Scale developed by Ryff.^33,34^ We used the 42-item version, which includes 7 items for each of the 6 following subconstructs of flourishing: purpose in life, self-acceptance, positive relations with others, personal growth, environmental mastery, and autonomy (eTable 2 in the Supplement). This scale is among the most widely used measures of eudaimonic well-being (ie, flourishing),^7,10,11^ and several studies have examined the association of this measure of flourishing with childhood family connection.^18,19,20,21,22,23^ Respondents rated each item on a Likert-type scale, ranging from strongly agree (1) to strongly disagree (7). Positively worded items were reverse-coded so that higher scores indicated greater flourishing. For each subconstruct score, we calculated the sum of the 7 items (range, 7-49). The overall flourishing score was the sum of all items across the 6 subconstructs (range, 42-294). The internal consistency (Cronbach α) of the flourishing score was 0.94 overall and at least 0.73 for each subconstruct (eTable 1 in the Supplement).

#### Measures of Childhood Adversity

We determined respondents’ exposure to 10 categories of ACEs related to abuse, neglect, and household challenges (eTable 3 in the Supplement)^35,36,37,38^ and counted (0-10) the number of categories of exposure. Childhood social position was derived from a validated question.^39^ Respondents were asked to consider their family’s social position while growing up and mark the family’s position (1-10) at a rung on the social ladder, with 10 being at the top rung (ie, “people who are the best off” in terms of money, schooling, and jobs) and 1 being at the bottom rung (ie, “people who are the worst off” in terms of money, schooling, and jobs). To facilitate interpretation of stratified analysis by level of childhood adversity, we used 3 levels of ACE exposure (ie, 0 categories, 1 category, and ≥2 categories) and childhood social position score (ie, low, 1-6; medium, 7-8; and high, 9-10).

#### Covariates

Respondents reported their age, sex, highest completed level of education, household income, and age at type 1 diabetes diagnosis. Data from separate questions on race and Hispanic ethnicity were combined to create 3 mutually exclusive race/ethnicity categories (ie, white, non-Hispanic; Hispanic, any race; other race, non-Hispanic). Hemoglobin A_1c_ (HbA_1c_) level was also treated as a covariate in our analysis. At the end of the survey fielding, the most recent HbA_1c_ level was abstracted from the NBDC medical record; HbA_1c_ level was assessed at NBDC visits using a DCA Vantage Analyzer point-of-care device (Siemens), which was calibrated 2 to 3 times per month.

### Statistical Analysis

Of the 423 respondents, we excluded 8 (1.9%) who were missing data on the family connection or flourishing measures, leaving an analytic sample of 415. We assessed the association of HbA_1c_ with family connection and flourishing scores using a Pearson correlation coefficient. Using *t* tests and 1-way analysis of variance, we assessed how the flourishing score was associated with levels of the 7 covariates and the 2 childhood adversity variables. To examine the association of family connection with flourishing, linear regression was used with flourishing as the dependent variable and family connection as the explanatory variable. When family connection was used in this model as a continuous variable, analysis of the standardized residuals suggested no evidence of a nonlinear association between family connection and flourishing. Unadjusted mean flourishing scores were determined for each tertile of family connection. For multivariable analyses, there was listwise deletion of 8 cases (1.9%) that were missing data on at least 1 of the covariates included in the regression model. Regression-based margins, standardized to the distribution of covariates in the study population, were used to estimate adjusted mean flourishing scores for each tertile of family connection. The standardized adjusted differences in flourishing scores between tertiles were determined by first standardizing the flourishing score (mean [SD], 0 [1]) and examining the regression coefficients for those with medium or high family connection relative to those with low family connection. The association between family connection and flourishing was also examined separately by levels of 2 variables related to childhood adversity, ie, ACE exposure (3 levels) and childhood social position (3 levels). A significance threshold of *P* < .05 from 2-sided testing was used.

## Results

The 423 survey respondents were a mean of 0.8 (95% CI, 0.3-1.3) years older than the 320 nonparticipants, and a greater percentage were female patients (251 [59.3%] vs 137 [42.8%]). Among survey respondents, the mean (SD) HbA_1c_ level was 8.0% (1.7%) compared with 8.3% (2.0%) among nonparticipants (to convert to proportion of total hemoglobin, multiply by 0.01).

The 415 participants (98.1%) included in the analysis had a mean (SD) age of 25.0 (3.2) years, with 246 (59.3%) female respondents, 288 (69.6%) non-Hispanic white respondents, and 266 (64.1%) respondents with a college or postgraduate degree (Table 1). The mean (SD) flourishing score was 221.8 (37.7) (Table 1; eTable 1 in the Supplement). There were no statistically significant differences in flourishing scores across levels of age, sex, race/ethnicity, or education. There was an inverse association between the family connection score and HbA_1c_ level (*r* = −0.13; *P* = .03) and between the flourishing score and HbA_1c_ level (*r* = −0.12; *P* = .02). Among the 143 participants (34.5%) with good glycemic control (ie, HbA_1c_ level ≤7.0%), the 181 (43.6%) with fair glycemic control (ie, HbA_1c_ level 7.1%-8.9%), and the 91 (21.9%) with poor glycemic control (ie, HbA_1c_ level ≥9%), the number of participants with flourishing scores in the upper quartile (ie, ≥252) were 45 (31.5%), 42 (23.2%), and 17 (18.7%), respectively (*P* = .08). Among the 104 participants with upper quartile flourishing scores, 59 (56.7%) did not have good glycemic control (ie, HbA_1c_ level >7.0%).

**Table 1.** Participant Characteristics and Their Association With Flourishing

Characteristic	No. (%)^a^	Flourishing Score	
		Mean (SD)	*P* Value^b^
All	415 (100)	221.8 (37.7)	NA
Age, y^c^			
19-21	88 (21.2)	223.6 (35.8)	.54
22-23	82 (19.8)	220.3 (38.8)	
24-25	87 (21.0)	218.5 (39.5)	
26-27	71 (17.1)	227.9 (38.1)	
28-31	87 (21.0)	219.6 (36.7)	
Sex			
Female	246 (59.3)	222.7 (36.3)	.54
Male	169 (40.7)	220.4 (39.8)	
Race/ethnicity			
White, non-Hispanic	288 (69.6)	222.2 (37.3)	.50
Hispanic, any race	96 (23.2)	223.4 (40.2)	
Other race, non-Hispanic	30 (7.2)	214.3 (33.2)	
Highest level of education			
≤High school diploma	29 (7.0)	209.4 (42.6)	.21
Some college or technical school	120 (28.9)	221.7 (38.7)	
College graduate	194 (46.8)	221.7 (36.6)	
Master’s or professional degree	72 (17.4)	227.0 (36.6)	
Household income, $			
<20 000	46 (11.3)	214.5 (38.9)	.01
20 000 to <40 000	62 (15.2)	209.4 (43.0)	
40 000 to <60 000	63 (15.5)	216.5 (32.0)	
60 000 to <80 000	73 (17.9)	230.1 (38.5)	
80 000 to <100 000	39 (9.6)	230.1 (35.4)	
100 000 to <150 000	56 (13.8)	226.3 (36.3)	
≥150 000	68 (16.7)	226.9 (37.0)	
Glycemic control^d^			
Good	143 (34.5)	227.4 (38.5)	.07
Fair	181 (43.6)	219.8 (38.0)	
Poor	91 (21.9)	216.8 (35.1)	
Age at type 1 diabetes diagnosis, y^e^			
0-4	52 (12.5)	221.0 (38.5)	.49
5-9	120 (28.9)	221.8 (36.9)	
10-14	129 (31.1)	217.9 (40.3)	
15-19	72 (17.4)	227.9 (32.8)	
≥20	42 (10.1)	224.2 (39.0)	
Categories of ACE exposure, No.^f^			
0	157 (37.8)	233.0 (32.6)	<.001
1	113 (27.2)	222.8 (39.4)	
≥2	145 (34.9)	208.8 (37.8)	
Childhood social position, score^g^			
High, 9-10	78 (18.9)	239.3 (29.3)	<.001
Medium, 7-8	187 (45.4)	221.5 (36.4)	
Low, 1-6	147 (35.7)	213.0 (40.5)	

Abbreviations: ACE, adverse childhood experience; NA, not applicable.

^a^ Percentages may not add to 100 because of rounding. Participants were missing data on characteristics as follows: race/ethnicity (1 [0.2%]), household income (8 [1.9%]), and childhood social position (3 [0.7%]).

^b^ *P* value is for *t* test or 1-way analysis of variance assessing how the flourishing score was associated with levels of a participant characteristic.

^c^ Describes age at the time of survey completion. The mean (SD [range]) age of the overall sample was 25.0 (3.2 [19.3-31.4]) years.

^d^ Based on hemoglobin A_1c_ values measured on a point-of-care device, which read to a maximum value of 14.0%; 5 participants (1.2%) had this value. Good glycemic control was defined as hemoglobin A_1c_ level of 7.0% or less; fair, 7.1% to 8.9%; and poor, 9.0% and higher. The mean (SD [range]) hemoglobin A_1c_ level of the overall sample was 8.0% (1.7% [5.2%-14.0%]) (to convert hemoglobin A_1c_ values to proportion of total hemoglobin, multiply by 0.01).

^e^ The mean (SD [range]) age at diagnosis of the overall sample was 11.2 (5.8 [0-28]) years.

^f^ Count of the 10 following categories of ACEs: emotional abuse, physical abuse, sexual abuse, emotional neglect, physical neglect, mother treated violently, parental separation or divorce, household substance abuse, household mental illness, and incarcerated household member.

^g^ See Methods section for details on scoring childhood social position.

After adjusting for covariates, mean flourishing scores increased from the lowest (201.0; 95% CI, 195.0-207.0) to medium (225.2; 95% CI, 219.4-231.0) to highest (240.4; 95% CI, 234.4-246.4) tertiles of family connection (Table 2). Compared with those in the lowest tertile of family connection, the adjusted flourishing scores were 1.04 (95% CI, 0.81-1.27) SDs higher among those in the highest tertile and 0.64 (95% CI, 0.42-0.86) SDs higher among those in the middle tertile. For each 1-SD increase in the family connection score, there was a 0.44 (95% CI, 0.34-0.53) SD increase in the adjusted flourishing score. Within each of the 3 subgroups determined by level of exposure to ACEs or childhood social position, there was also a significant, graded association between family connection and flourishing (Figure; eTable 4 in the Supplement). In each of the 6 subgroups, the adjusted flourishing score increased significantly across tertiles of family connection. For these subgroups, the flourishing score was between 0.70 (95% CI, 0.22-1.18) and 1.58 (95% CI, 1.15-2.01) SDs higher in the highest tertile of family connection compared with the lowest. For example, in the subgroup of participants with 2 or more ACEs, those in the highest tertile of family connection had adjusted flourishing scores 0.76 (95% CI, 0.14-1.38) SD units higher than those in the lowest tertile. In the subgroup of participants with low childhood social position, those in the highest tertile of family connection had flourishing scores 1.08 (95% CI, 0.63-1.52) SD units higher than those in the lowest tertile.

**Table 2.** Association of Family Connection With Flourishing Among 407 Participants^a^

Family Connection Tertile	No. (%)	Flourishing Score			
		Mean (95% CI)		Difference (95% CI)	
		Unadjusted	Adjusted^b^	Adjusted^b^^,^^c^	Standardized Adjusted^b^^,^^d^
Low, <3.13	139 (34.2)	200.9 (195.2-206.6)	201.0 (195.0-207.0)	0 [Reference]	0 [Reference]
Medium, 3.13-3.70	143 (35.1)	225.1 (219.4-230.7)	225.2 (219.4-231.0)	24.2 (15.7-32.6)	0.64 (0.42-0.86)
High, >3.70	133 (32.7)	240.0 (234.2-245.9)	240.4 (234.4-246.4)	39.4 (30.7-48.1)	1.04 (0.81-1.27)

^a^ There was listwise deletion of 8 participants who were missing data on 1 or more of the covariates included in the regression model.

^b^ Adjusted for the following variables: age (continuous), sex, race/ethnicity, highest level of participant education, household income, age at type 1 diabetes diagnosis (continuous), and hemoglobin A_1c_ level (continuous).

^c^ Obtained from the *B* coefficients for the dummy variables in the regression representing the medium and high tertiles of family connection, using the raw flourishing score as the dependent variable.

^d^ Obtained from the β coefficients for the dummy variables in the regression representing the medium and high tertiles of family connection, using the standardized flourishing score as the dependent variable.

**Figure.**
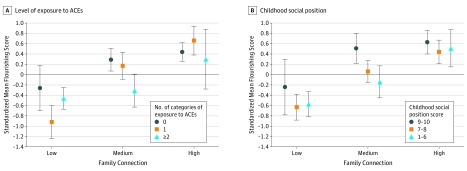
Association of Family Connection With Flourishing by Levels of Childhood Adversity All points represent standardized mean flourishing scores, and the whiskers represent 95% CIs. Standardized means and 95% CIs for each subgroup were derived from a separate regression model for a given subgroup (ie, a total of 6 models). Each model estimated the standardized mean flourishing score at high, medium, and low tertiles of family connection, adjusting for the following variables: age (continuous), sex, race/ethnicity, highest level of education, household income, age at type 1 diabetes diagnosis (continuous), and hemoglobin A_1c_ level (continuous). Additional details on the results appear in eTable 4 in the Supplement. See the Methods section for descriptions of adverse childhood experiences (ACEs) and childhood social position.

After adjusting for covariates, there was a significant association between family connection and the scores for all 6 subconstructs of flourishing (eg, purpose in life: β = 0.33; 95% CI, 0.23-0.43; *P* < .001; self-acceptance: β = 0.43; 95% CI, 0.34-0.53; *P* < .001) (eTable 5 in the Supplement). When maternal and paternal connection scores were analyzed separately, the associations between connection and the flourishing score were both significant and of similar magnitude. Compared with those in the lowest tertile of maternal connection, the adjusted flourishing scores were 0.89 (95% CI, 0.65-1.13) SD units higher among those in the highest tertile. The adjusted flourishing scores were 0.99 (95% CI, 0.75-1.25) SD units higher among those in the highest tertile of paternal connection compared with those in the lowest tertile (eTable 6 in the Supplement).

## Discussion

In a cross-sectional survey of young adults receiving specialty care for type 1 diabetes, higher levels of remembered, childhood family connection were associated with greater flourishing in young adulthood. The graded association between family connection and flourishing remained after adjusting for covariates, including current glycemic control, age at type 1 diabetes diagnosis, level of education, and income. A similar graded association was also present across levels of ACEs and childhood social position. Flourishing and family connection were only weakly associated with glycemic control.

We are aware of 6 studies examining the association of childhood family connection with adult flourishing that measured flourishing, as we did, with the Psychological Well-being Scale developed by Ryff.^18,19,20,21,22,23^ None of these studies examined this association in the context of childhood-onset chronic disease. Four of the studies used data from MIDUS, a large population-based study of US adults aged 25 to 74 years; these studies used, as we did, a measure of family connection based on items in MIDUS.^18,19,22,23^ Each study demonstrated a significant association between childhood family connection and adult flourishing. The magnitude of these associations was weaker than in our study, but the adults we studied were younger and likely had fewer life experiences that could affect flourishing independent of childhood family connection. A fifth study, using data from the 1946 British Birth Cohort,^20^ observed women over time and showed a significant association between recalled parental care and flourishing at age 52 years. Finally, among a sample of men from the Veterans Affairs Normative Aging Study,^21^ those who reported childhood experiences characterized as “cherished,” including close family relationships, had higher levels of flourishing in later adulthood (mean age of 75.5 years) than those reporting harsher or more neutral childhood experiences.

We did not evaluate possible mechanisms to explain the association of childhood family connection with adult flourishing. Others have shown that this association may be mediated by factors assessed in adulthood, such as extraversion,^20^ generativity and the experience of parenting one’s own offspring,^18^ a problem-focused coping style,^22^ and social support.^21^ Some of the mechanisms may also involve an adaptive psychobiological response to stress.^40,41^ A cross-sectional study of US children^30^ showed that family connection was associated with an index of child flourishing based on self-regulation, persistence, and openness, which may be precursors of adult flourishing. However, to our knowledge, there have been no studies associating this index of child flourishing with adult flourishing.

We can only speculate about the biobehavioral mechanisms that may cause childhood family connection to result in adult flourishing. Healthy relationships between adult caregivers and children may lead to optimal integration of neural networks during development. These integrated connections include those between the brain’s hemispheres,^42^ between the limbic and cortical regions of the brain,^43^ and between the brain and body.^44^ In those who flourish, we speculate that their physical health will be reflected in adaptive immune responses, in both the brain and body,^45^ and that their behavioral health will be reflected in a relational awareness and responsiveness to other humans, to one’s self, and to the nonhuman environment.^46^

Eudaimonic well-being in adulthood, what we have here called flourishing, is a potentially valuable outcome to consider in pediatric care. A 2011 consensus definition describes health as “the ability to adapt and self-manage in the face of social, physical, and emotional challenges.”^5^ Health, as flourishing with adversity, is an extension of this definition to consider.^47^ This concept extends the traditional notion of resilience from surviving after difficulty to thriving.^13^ It includes the possibility that adversities, which all children will eventually face, can contribute to flourishing and that flourishing occurs when adversity is combined with adult support, as reflected in family connection. If health is conceptualized as flourishing with adversity, this might encourage pediatricians, as a complement to their primary role in disease prevention and treatment, to also emphasize factors like family connection, which may lead to adult flourishing. Pediatric health care involves helping children and parents deal with suffering and loss, while still embracing core dimensions of adult flourishing, ie, growth with the maximization of one’s potential in relation to changing abilities and limitations. This approach to care, while acknowledging and addressing remediable adversities,^16^ can help focus on patient and family goals and assets.^48^

### Limitations

This study has limitations. In a cross-sectional analysis, we cannot infer a causal relationship between childhood family connection and adult flourishing, and the association we demonstrated is potentially influenced by common rater bias.^49^ Data suggest that recall of positive parental relations is likely to be accurate,^50,51,52^ but we cannot exclude the possibility that adults with greater flourishing, owing to factors unrelated to childhood events, may be biased in recalling closer family connection. Our results cannot necessarily be generalized to other young adults with childhood-onset chronic diseases or to all young adults with type 1 diabetes. Survey respondents had successfully sought specialty care for their type 1 diabetes, and the association between family connection and flourishing may have differed between those we studied and those not receiving specialty care or not responding to the survey. Those who participated in the survey were disproportionately female patients and had better glycemic control than those who did not participate.

In this article, we used the term flourishing as it was originally applied to eudaimonic well-being^12^ and as operationalized in our measure (eTable 2 in the Supplement). However, this is not the only scale of eudaimonic well-being,^10^ and different associations between family connection and flourishing might be seen with other measures of flourishing. For example, the term flourishing has been used more recently to describe measures of adult well-being that combine the subjective components of eudaimonic and hedonic well-being^53,54,55^ or also include the objective components of physical health and economic sufficiency.^56^ We use the term flourishing more narrowly here to build on the existing literature that associates eudaimonic well-being with childhood family connection^18,19,20,21,22,23^ and to chronic health conditions.^1,2,3,4,57,58^

## Conclusions

In this cross-sectional study of young adults with type 1 diabetes, higher levels of childhood family connection were associated with greater flourishing in young adulthood across levels of childhood adversity. Supporting family connection is resonant with a core goal of pediatrics, namely to promote optimal social-emotional development in children, and we have shown how childhood family connection may also be associated with the outcome of adult flourishing. In contrast to showing how a lack of childhood family connection is associated with later problems, we have shown how its presence may promote adult flourishing. These data provide an optimistic rationale for increasing attention to family connection, especially in the setting of chronic illness and other adversity.

## Notes

Supplement. **eAppendix. **Supplemental Methods Description **eTable 1. **Sample Descriptive Statistics and Internal Consistency for the Flourishing Score, Subconstruct Scores, and Family Connection Scores **eTable 2. **Description of Flourishing (Eudaimonic Well-being) Subconstructs **eTable 3. **Categories of Adverse Childhood Experiences, Survey Questions, and Response Categorizations to Determine Exposure **eTable 4. **Association of Family Connection With Flourishing by Level of Childhood Adversity **eTable 5. **Linear Regression Models of Family Connection Score Predicting Flourishing Score and Subconstruct Scores **eTable 6. **Association of Maternal and Paternal Connection With Flourishing **eReferences.**
