# Supplementary material for: Association of Childhood Family Connection With Flourishing in Young Adulthood Among Those With Type 1 Diabetes
Source: JAMA Netw Open. 2020 Mar 5;3(3):e200427. doi: 10.1001/jamanetworkopen.2020.0427 (PMC7059021; doi:10.1001/jamanetworkopen.2020.0427)
Supplement: Supplement. — eAppendix. Supplemental Methods Description eTable 1. Sample Descriptive Statistics and Internal Consistency for the Flourishing Score, Subconstruct Scores, and Family Connection Scores eTable 2. Description of Flourishing (Eudaimonic Well-being) Subconstructs eTable 3. Categories of Adverse Childhood Experiences, Survey Questions, and Response Categorizations to Determine Exposure eTable 4. Association of Family Connection With Flourishing by Level of Childhood Adversity eTable 5. Linear Regression Models of Family Connection Score Predicting Flourishing Score and Subconstruct Scores eTable 6. Association of Maternal and Paternal Connection With Flourishing eReferences. [file jamanetwopen-3-e200427-s001.pdf]

Supplementary Online Content

Whitaker RC, Dearth-Wesley T, Herman AN, Nagel KE, Smith HG, Weil HFC. Association of childhood family connection with flourishing in young adulthood among those with type 1 diabetes. *JAMA Netw Open*. 2020;3(3):e200427. doi:10.1001/jamanetworkopen.2020.0427

eAppendix. Supplemental Methods Description

eTable 1. Sample Descriptive Statistics and Internal Consistency for the Flourishing Score, Subconstruct Scores, and Family Connection Scores

eTable 2. Description of Flourishing (Eudaimonic Well-being) Subconstructs

eTable 3. Categories of Adverse Childhood Experiences, Survey Questions, and Response Categorizations to Determine Exposure

eTable 4. Association of Family Connection with Flourishing by Level of Childhood Adversity

eTable 5. Linear Regression Models of Family Connection Score Predicting Flourishing Score and Subconstruct Scores

eTable 6. Association of Maternal and Paternal Connection with Flourishing

eReferences.

This supplementary material has been provided by the authors to give readers additional information about their work.

© 2020 Whitaker RC et al. *JAMA Network Open.*

eAppendix. Supplemental Methods Description

Detailed survey procedures used in "T1 Flourish: Strengths and Challenges for Young Adults with Type 1 Diabetes"

Setting

Study participants were recruited from a diabetes specialty clinic at the Naomi Berrie Diabetes Center (NBDC) at the Columbia University Irving Medical Center, which is a tertiary care medical center serving the greater New York City metropolitan area.

Eligible participants

Eligible participants were all patients with a diagnosis of type 1 diabetes who had at least one outpatient visit to the NBDC clinic between December 1, 2015 and November 30, 2016 and were age 18.0 to <30.0 years on December 1, 2015. The 746 potentially eligible patients identified through electronic administrative records were reviewed by NBDC physicians. Three patients were excluded because they had cognitive impairments that would have prevented them from comprehending the survey, leaving 743 eligible participants.

Survey content

During the spring of 2017 (February 13, 2017 through June 30, 2017), all 743 eligible participants with type 1 diabetes were given the opportunity to complete an online survey called "T1 Flourish: Strengths and Challenges for Young Adults with Type 1 Diabetes." The survey contained questions on sociodemographics, adverse childhood experiences (ACEs), and diabetes clinical history, as well as validated scales assessing diabetes-related distress, dispositional mindfulness, and aspects of positive psychological well-being. The survey was administered using Qualtrics software (Qualtrics Labs, Inc., Provo, Utah), was available in English and Spanish, and took approximately 30 minutes to complete.

Survey fielding

The 743 eligible patients received a letter via email or posted mail from their treating physician at NBDC inviting them to participate in the survey. Flyers announcing the survey were also posted at the NBDC clinic and on its website. All recruitment materials were provided in English and Spanish.

Interested, eligible patients accessed the survey at the study's website after providing authenticating information (last name and birthdate) and informed consent online. Participants could complete the survey on any computer, tablet, or smartphone with internet access. All online respondents who completed the survey were sent an electronic $20 Amazon gift card. All eligible patients who visited the NBDC clinic during the data collection period were reminded of the survey by clinic staff and given the opportunity to complete the survey using a tablet in clinic. Those who completed the survey in the clinic had the option of receiving their $20 compensation in cash.

During the first month of data collection, survey non-respondents received weekly email reminders about the survey. In the final two months of data collection, non-respondents received a minimum of two phone calls from study staff. For any non-respondent reached by phone, follow-up reminder text messages were sent to those who agreed to receive them. In the final month of recruitment, treating physicians were given weekly updates of their non-responding patients and had the opportunity to contact these patients by phone about the survey. During the final two weeks of data collection, all non-respondents were sent three "last call" emails signed by the treating physician.

© 2020 Whitaker RC et al. *JAMA Network Open.*

eTable 1. Sample Descriptive Statistics and Internal Consistency for the Flourishing Score, Subconstruct Scores, and Family Connection Scores

Construct/Subconstruct	*n*	Mean/ Median	*SD/IQR*	Range	Cronbach Alpha	MIDUS Mean/Median (*SD/IQR*)[c]
Flourishing	415	221.8	37.7	111 - 291	.94	226.8 (33.2)
Purpose in life	415	37.9	7.5	16 - 49	.76	38.5 (6.5)
Self-acceptance	415	35.1	9.4	8 - 49	.87	37.0 (8.1)
Positive relations	415	39.7	7.8	15 - 49	.82	39.7 (7.0)
Personal growth	415	40.7	6.3	16 - 49	.75	39.3 (6.3)
Environmental mastery	415	33.5	8.7	9 - 49	.83	36.4 (7.4)
Autonomy	415	34.8	7.4	11 - 49	.73	36.0 (7.0)
Family connection[a]	415	3.4	(2.9, 3.8)	1 - 4	.92	3.1 (2.6, 3.5)
Maternal connection	415	3.7	(3.2, 4.0)	1 - 4	.90	3.3 (2.8, 3.7)
Paternal connection[b]	395	3.3	(2.6, 3.8)	1 - 4	.93	2.9 (2.2, 3.5)

NOTE: *SD* = standard deviation, *IQR* = interquartile range

[a] The family connection score was determined by averaging the maternal and paternal connection scores. For the 20 respondents who reported not having a father or man who raised them, the maternal connection score was used.

[b] Twenty participants responded “Did not have a father or man who raised me” to the following question: “How would you rate your relationship with your father (or the man who raised you) during the years you were growing up?” These participants were not asked to complete the other 6 paternal connection items and did not receive a paternal connection score.

[c] For comparison purposes, the mean/median (*SD/IQR*) are shown for each construct/subconstruct for participants from the public use data files of the Midlife in the United States Study (MIDUS) (available at http://www.midus.wisc.edu/data/index.php). MIDUS participants completed the 7-item childhood maternal and paternal connection measures in MIDUS 1 (1995-1996) and the 42-item version of Ryff’s Psychological Well-being Scale (flourishing) in MIDUS 2 (2004-2006). The longitudinal sample includes the participants 25 to 31 years of age in MIDUS 1 with complete family connection and flourishing measures (n = 350).

© 2020 Whitaker RC et al. *JAMA Network Open.*

eTable 2. Description of Flourishing (Eudaimonic Well-being) Subconstructs

**Subconstruct**	**Description**
**Purpose in life**	
High scorer	Has goals in life and a sense of directedness; feels there is meaning to present and past life; holds beliefs that give life purpose; has aims and objectives for living
Low scorer	Lacks a sense of meaning in life; has few goals or aims, lacks sense of direction; does not see purpose in past life; has no outlooks or beliefs that give life meaning
**Self-acceptance**	
High scorer	Possesses a positive attitude toward the self; acknowledges and accepts multiple aspects of self, including good and bad qualities; feels positive about past life
Low scorer	Feels dissatisfied with self; is disappointed with what has occurred in past life; is troubled about certain personal qualities; wishes to be different than what he or she is
**Positive relations with others**	
High scorer	Has warm, satisfying, trusting relationships with others; is concerned about the welfare of others; capable of strong empathy, affection and intimacy; understands give and take of human relationships
Low scorer	Has few close, trusting relationships with others; finds it difficult to be warm, open, concerned about others; is isolated and frustrated in interpersonal relationships; not willing to make compromises to sustain important ties with others
**Personal growth**	
High scorer	Has a feeling of continued development; sees self as growing and expanding; is open to new experiences; has sense of realizing his or her potential; sees improvement in self and behavior over time; is changing in ways that reflect more self-knowledge and effectiveness
Low scorer	Has a sense of personal stagnation; lacks sense of improvement or expansion over time; feels bored and uninterested with life; feels unable to develop new attitudes or behaviors
**Environmental mastery**	
High scorer	Has a sense of mastery and competence in managing the environment; controls complex array of external activities; makes effective use of surrounding opportunities; able to choose or create contexts suitable to personal needs and values
Low scorer	Has difficulty managing everyday affairs; feels unable to change or improve surrounding context; is unaware of surrounding opportunities; lacks sense of control over external world
**Autonomy**	
High scorer	Is self-determining and independent; able to resist social pressures to think and act in certain ways; regulates behavior from within; evaluates self by personal standards
Low scorer	Is concerned about the expectations and evaluations of others; relies on judgments of others to make important decisions; conforms to social pressures to think and act in certain ways

Adapted with permission from S. Karger AG (Basel, Switzerland) as published in Ryff CD. Psychological well-being revisited: advances in the science and practice of eudaimonia. *Psychother Psychosom*. 2014;83:10-28. doi: 10.1159/000353263

eTable 3. Categories of Adverse Childhood Experiences, Survey Questions, and Response Categorizations to Determine Exposure

Category of ACE	Question	Response Categorization
**Emotional abuse**	How often did a parent or adult in your home ever swear at you, insult you, or put you down?	Yes (more than once); No (once, never)
**Physical abuse**	Before age 18, how often did a parent or adult in your home ever hit, beat, kick, or physically hurt you in any way? Do not include spanking.	Yes (once, more than once); No (never)
**Sexual abuse**	*Yes (if yes to any of the three questions below); No (if no to all three questions)*	
	How often did anyone at least 5 years older than you or an adult, ever touch you sexually?	Yes (once, more than once); No (never)
	How often did anyone at least 5 years older than you or an adult try to make you touch them sexually?	Yes (once, more than once); No (never)
	How often did anyone at least 5 years older than you or an adult force you to have sex?	Yes (once, more than once); No (never)
**Emotional neglect**	*Yes (if yes to either question below); No (if no to both questions)*	
	How often did you feel that no one in your family loved you or thought you were important or special?	Yes (often, very often); No (never, sometimes)
	How often did you feel that your family didn't look out for each other, feel close to each other, or support each other?	Yes (often, very often); No (never, sometimes)
**Physical neglect**	*Yes (if yes to either question below); No (if no to both questions)*	
	How often did you feel that you didn't have enough to eat, had to wear dirty clothes, and had no one to protect you?	Yes (often, very often); No (never, sometimes)
	Before age 18, how often did you feel that your parents were too drunk or high to take care of you or take you to the doctor if you needed it?	Yes (often, very often); No (never, sometimes)
**Household mental illness**	Did you live with anyone who was depressed, mentally ill, or suicidal?	Yes; No
**Incarcerated household member**	Did you live with anyone who served time or was sentenced to serve time in a prison, jail, or other correctional facility?	Yes; No
**Household substance abuse**	*Yes (if yes to either question below); No (if no to both questions)*	
	Did you live with anyone who was a problem drinker or alcoholic?	Yes; No
	Did you live with anyone who used illegal street drugs or who abused prescription medications?	Yes; No
**Parental separation or divorce**	Were your parents separated or divorced?	Yes; No (no, parents not married)
**Intimate partner violence**	How often did your parents or adults in your home ever slap, hit, kick, punch, or beat each other up?	Yes (once, more than once); No (never)

Note: The preamble to the above set of questions asks respondents to reference the time period before 18 years of age; two items also state "Before age 18" in the question. Two participants were missing data on all 3 sexual abuse questions and for the analyses were assumed to have no exposure to sexual abuse. Eleven of the questions, covering eight categories of adversity, were those used in the Centers for Disease Control and Prevention Behavioral Risk Factor Surveillance System ACEs module.[1,2] Four other questions asked about the two additional categories of emotional and physical neglect. These questions were adapted from the "What's Your ACE Score?" questionnaire.[3] The 4 questions were based on 10-items in the Childhood Trauma Questionnaire used to assess emotional and physical neglect in the ACEs Study conducted in the Kaiser Health Plan in San Diego, CA.[4]

© 2020 Whitaker RC et al. *JAMA Network Open.*

eTable 4. Association of Family Connection With Flourishing by Level of Childhood Adversity

Exposure Group				Flourishing Score			
**Level of Childhood Adversity**	**Family Connection Tertile**	**No. in Group**		**Unadjusted Mean (95% CI)**	**Adjusted Mean (95% CI)[a]**	**Adjusted Difference (95% CI)[a,b]**	**Standardized Adjusted Difference (95% CI)[a,c]**
**ACEs[d]**							
None							
	Low	16		209.1 (193.6, 224.6)	211.7 (195.3, 228.2)	Reference	Reference
	Medium	59		230.1 (222.0, 238.2)	232.6 (224.4, 240.8)	20.8 (2.0, 40.0)	0.55 (0.05, 1.05)
	High	82		239.6 (232.8, 246.5)	238.3 (231.4, 245.2)	26.5 (8.5, 44.5)	0.70 (0.22, 1.18)
1							
	Low	30		188.7 (176.8, 200.7)	186.9 (174.9, 198.8)	Reference	Reference
	Medium	45		227.6 (217.8, 237.4)	228.3 (218.4, 238.2)	41.4 (25.5, 57.4)	1.10 (0.68, 1.52)
	High	38		244.2 (233.5, 254.8)	246.6 (236.1, 257.0)	59.7 (43.5, 75.8)	1.58 (1.15, 2.01)
2 or more							
	Low	93		203.4 (195.8, 211.0)	204.6 (196.8, 212.3)	Reference	Reference
	Medium	39		214.6 (202.8, 226.4)	209.9 (197.9, 221.9)	5.3 (-9.3, 19.9)	0.14 (-0.25, 0.53)
	High	13		230.2 (209.8, 250.5)	233.2 (211.4, 254.9)	28.6 (5.1, 52.0)	0.76 (0.14, 1.38)
**Childhood social position[e]**							
High (9-10)							
	Low	12		216.1 (200.0, 232.2)	212.5 (192.1, 232.9)	Reference	Reference
	Medium	26		243.1 (232.2, 254.0)	241.1 (229.9, 252.2)	28.6 (3.9, 53.2)	0.76 (0.10, 1.41)
	High	40		243.8 (235.1, 252.7)	245.5 (236.7, 254.2)	33.0 (9.8, 56.2)	0.87 (0.26, 1.49)
Medium (7-8)							
	Low	53		198.0 (189.1, 206.9)	197.9 (188.5, 207.4)	Reference	Reference
	Medium	74		223.9 (216.4, 231.4)	224.2 (216.5, 232.0)	26.3 (13.9, 38.7)	0.70 (0.37, 1.03)
	High	60		239.4 (231.1, 247.8)	238.4 (229.8, 247.2)	40.5 (27.4, 53.6)	1.07 (0.73, 1.42)
Low (1-6)							
	Low	74		200.6 (191.9. 209.3)	200.2 (191.4, 209.2)	Reference	Reference
	Medium	42		216.6 (205.0, 228.2)	216.4 (204.5, 229.3)	16.2 (1.0, 31.4)	0.43 (0.03, 0.83)
	High	31		237.7 (224.2, 251.2)	240.9 (227.2, 254.6)	40.6 (23.9, 57.4)	1.08 (0.63, 1.52)

© 2020 Whitaker RC et al. *JAMA Network Open.*

[a] N = 407. There was listwise deletion of 8 cases that were missing data on one or more of the covariates included in the regression models. Adjusted for the following variables: age (continuous), sex, race/ethnicity, highest level of education, household income, age at type 1 diabetes diagnosis (continuous), and HbA_{1c} level (continuous).

[b] Obtained from the beta coefficients for the dummy variables in the regression representing the medium and high tertiles of family connection, using the raw flourishing score as the dependent variable.

[c] Obtained from the beta coefficients for the dummy variables in the regression representing the medium and high tertiles of family connection, using the standardized flourishing score as the dependent variable.

[d] Count of 10 categories of adverse childhood experiences (ACEs). See eTable 3.

[e] Based on childhood social position score (1-10). Childhood social position score was derived from the following question, provided with an image of a 10-rung ladder: "Imagine that this ladder pictures how American society is set up. At the top of the ladder are the people who are the best off – they have the most money, the highest amount of schooling, and the jobs that bring the most respect. At the bottom are the people who are the worst off – they have the least money, little or no education, no job or jobs that no one wants or respects. Now think about your family. When you were growing up in your family, where do you think your family would have been on this ladder? Indicate the number that best represents where your family would have been on this ladder." (Scores could range from 1 [worst off] to 10 [best off]).

© 2020 Whitaker RC et al. *JAMA Network Open.*

eTable 5: Linear Regression Models of Family Connection Score Predicting Flourishing Score and Subconstruct Scores

**Dependent Variable**	**Standardized Beta (95% CI)**	***P* value**
Flourishing	0.44 (0.34, 0.53)	<.001
Purpose in life	0.33 (0.23, 0.43)	<.001
Self-acceptance	0.43 (0.34, 0.53)	<.001
Positive relations with others	0.50 (0.41, 0.60)	<.001
Personal growth	0.32 (0.22, 0.42)	<.001
Environmental mastery	0.37 (0.27, 0.47)	<.001
Autonomy	0.11 (0.01, 0.22)	.038

Note: Separate linear regression models were run for each dependent variable (7 models total). In each model, the family connection score and the dependent variable were standardized (M = 0, SD = 1). The standardized beta coefficient represents the magnitude of the increase (in standard deviations) of the dependent variable for each 1 SD increase in family connection score. Each model adjusts for the following variables: age (continuous), sex, race/ethnicity, highest level of education, household income, age at type 1 diabetes diagnosis (continuous), and HbA_{1c} level (continuous). N = 407 for each regression model. There was listwise deletion of 8 cases that were missing data on one or more of the covariates included in the regression model.

© 2020 Whitaker RC et al. *JAMA Network Open.*

eTable 6: Association of Maternal and Paternal Connection With Flourishing

**Exposure Group**			**Flourishing Score**			
**Connection tertile**	**No. in Tertile**		**Unadjusted Mean (95% CI)**	**Adjusted Mean (95% CI)[a]**	**Adjusted Difference (95% CI)[a,b]**	**Standardized Adjusted Difference (95% CI)[a,c]**
Maternal[d]						
Low (<3.35)	139		204.6 (198.8, 210.5)	204.7 (198.6, 210.8)	Reference	Reference
Medium (3.35-3.86)	153		224.1 (218.5, 229.7)	224.5 (218.9, 230.2)	19.8 (11.5, 28.2)	0.53 (0.30, 0.75)
High (>3.86)	123		238.2 (231.9, 244.5)	238.3 (232.0, 244.7)	33.6 (24.7, 42.6)	0.89 (0.65, 1.13)
Paternal[e]						
Low (<2.96)	133		203.5 (197.6, 209.4)	204.9 (198.6, 211.2)	Reference	Reference
Medium (2.96-3.68)	141		221.0 (215.3, 226.8)	219.9 (213.9, 225.9)	15.0 (6.0, 24.0)	0.40 (0.16, 0.64)
High (>3.68)	121		241.7 (235.5, 247.9)	242.3 (235.9, 248.7)	37.4 (28.2, 46.6)	0.99 (0.75, 1.24)

[a] Adjusted for the following variables: age (continuous), sex, race/ethnicity, highest level of participant education, household income, age at type 1 diabetes diagnosis (continuous), and HbA_{1c} level (continuous).

[b] Obtained from the beta coefficients for the dummy variables in the regression representing the medium and high tertiles of family connection, using the raw flourishing score as the dependent variable.

[c] Obtained from the beta coefficients for the dummy variables in the regression representing the medium and high tertiles of family connection, using the standardized flourishing score as the dependent variable.

[d] N = 407. There was listwise deletion of 8 cases that were missing data on one or more of the covariates included in the regression model.

[e] N = 387. Twenty participants responded “Did not have a father or man who raised me” to the following question: “How would you rate your relationship with your father (or the man who raised you) during the years you were growing up?” These participants were not asked to complete the other 6 paternal connection items and did not receive a paternal connection score. There was listwise deletion of an additional 8 cases that were missing data on one or more of the covariates included in the regression model.

© 2020 Whitaker RC et al. *JAMA Network Open.*

eReferences

1. Bynum L, Griffin T, Riding D, et al. Adverse childhood experiences reported by adults-five states, 2009. *Morb Mortal Weekly Rep*. 2010;59(49):1609-1613.

2. Centers for Disease Control and Prevention. Violence prevention: about Behavioral Risk Factor Surveillance System ACE Data [web page]. https://www.cdc.gov/violenceprevention/acestudy/ace_brfss.html. Accessed October 24, 2019.

3. Stevens JE. Got your ACE score? [on the Internet]. https://acestoohigh.com/got-your-ace-score/. Accessed October 24, 2019.

4. Dong M, Anda RF, Felitti VJ, et al. The interrelatedness of multiple forms of childhood abuse, neglect, and household dysfunction. *Child Abuse Negl*. 2004;28(7):771-784. doi: 10.1016/j.chiabu.2004.01.00

© 2020 Whitaker RC et al. *JAMA Network Open.*
